# *In vitro* culture of mechanically isolated murine primary follicles in the presence of human platelet lysate PLTMax

**DOI:** 10.5935/1518-0557.20240008

**Published:** 2024

**Authors:** Vera Lucia Lângaro Amaral, Jhuly Laurentino Nunes, Rafael Alonso Salvador, Alfred Paul Senn, Tiago Góss dos Santos

**Affiliations:** 1 Laboratory of reproductive biology, University of Vale do Itajaí (UNIVALI), Itajaí, SC, Brazil; 2 Department of Genetic Medicine and Development, University of Geneva, Geneva, Switzerland; 3 A.C. Camargo Cancer Center, São Paulo, SP, Brazil

**Keywords:** murine, oocytes, *in vitro* maturation, 3D follicle culture, PLTMax

## Abstract

**Objective:**

To develop a system for the culture of murine preantral ovarian follicles using Human Serum Albumin (HSA) and Human Platelet Lysate (PLTMax).

**Methods:**

Mechanically isolated preantral follicles (N=146) were obtained from Swiss mice and cultured in DMEM:F12 medium for ten days in a 96-well plate with conical bottom. The medium was supplemented with penicillin, streptomycin, and equine chorionic gonadotropin. Additional proteins were tested in 4 test groups: G1: human serum albumin (HSA), G2: human platelet lysate (PLTM), and G3 and G4: HSA + PLTMax at lower and higher concentrations, respectively. Cellular vitality and oocyte morphology were evaluated on day 11 of culture.

**Results:**

The highest follicular growth (3.4 fold) was achieved in HSA (G1), while a significantly lower (1.8 fold) growth was achieved in the presence of PLTM (G2, G4) and even further reduced (1.2 fold) when HSA and PLTM were combined (G3). Cellular vitality was close to 70-80% among the four groups, and the highest number of intact oocytes were found in G1.

**Conclusions:**

PLTM did not improve follicular development and oocyte maturation compared to HSA but preserved cell vitality.

## INTRODUCTION

Cancer remains one of the world’s most serious health problems and a leading cause of death (Donnez & Dolmans, 2018). The treatment of neoplasms with chemotherapy and radiotherapy has significantly increased patient survival. However, these treatments are aggressive to children or women of childbearing age as they have a cytotoxic action on the ovaries and pose a high risk to their function (Donnez & Dolmans, 2018). Because of this gonadotoxicity, strategies are being researched to preserve patient fertility (Donnez & Dolmans, 2015; Suzuki *et al.*, 2015; Lee *et al.*, 2021). Oncofertility is a recent medical specialty that combines oncology and reproductive endocrinology to preserve patients’ reproductive function (Santaballa *et al.*, 2022).

Already well-established techniques, such as oocyte and embryo cryopreservation, have been used to preserve fertility (Prentice *et al.*, 2011; Silber & Barbey, 2012). However, cryopreservation of ovarian tissue is still considered the only alternative capable of ensuring fertility in prepubertal women in the process of ovarian failure by cancer treatment (Silber & Barbey, 2012). A serious concern associated with this approach is the risk of reimplanting malignant cells along with the transplanted tissue, especially in patients with leukemia, the most common hematologic cancer in women under 20 (Jadoul *et al.*, 2012).

The development of isolated ovarian follicles *in vitro* is a promising alternative for preserving female fertility. Culture can be performed before or after cryopreservation of ovarian tissue, and mature oocytes can be obtained, as described in studies in mice (Jin *et al.*, 2010), monkeys (Xu *et al.*, 2013; Xiao *et al.*, 2015), and humans (Xiao *et al.*, 2015; McLaughlin *et al.*, 2018; Xu *et al.*, 2021).

Follicles can be inserted *in situ* in the tissue itself or cultured as an isolated form in a two-dimensional or three-dimensional system, where in the latter, follicles are included in a 3D matrix formed by a biopolymer (Rajabzadeh *et al.*, 2015; Figueiredo & Lima, 2017). In two-dimensional culture, the follicular structure is lost due to its adhesion to the culture dish or the embedding substrate (Asaduzzman *et al.*, 2018). However, using V-bottom culture plates, the maintenance of follicular architecture can be achieved (Telfer & Zelinski, 2013; Nikiforov *et al.*, 2018). For follicular development, the culture medium should be supplemented with gonadotropins such as FSH, protein such as HSA (human serum albumin) or FBS (fetal bovine serum), mitogenic and antioxidant agents such as ITS (insulin, transferrin, and selenium), antibiotics, and antifungals such as streptomycin and penicillin (Xiao *et al.*, 2015; Yin *et al.*, 2016; Amoushahi *et al.*, 2017; Mesalam *et al.*, 2019).

Human platelet lysate (hPL) has recently been used as a potential protein supplement for *in vitro* cell culture of bone marrow cells, adipose tissue, and mesenchymal stem cells, among others. Platelets are a natural reservoir of many proteins, cytokines, and growth factors (GFs). Extraction of GFs by lysis of human platelets represents a cheaper and safer alternative to using recombinant GFs or animal proteins (Burnouf *et al.*, 2016; Santos *et al.*, 2018). Some regulatory factors of folliculogenesis are present in platelets, such as epidermal growth factor (EGF), which ensures the proliferation of granulosa and theca cells (Magalhães-Padilha *et al.*, 2012; Jones & Shikanov, 2019), and insulin-like growth factor-1 (IGF-1), which is involved in the development of follicles at pre-antral stages (Magalhães-Padilha *et al.*, 2012), among others.

The commercial human platelet lysate (PLTMax^®^, PLTM, PL Bioscience, Aachen Germany) is a supplement rich in proteins and growth factors and has been used for cell proliferation *in vitro*, being a potential substitute for fetal bovine serum or another similar protein source (Alonso-Camino & Mirsch, 2019; Kakudo *et al.*, 2019). However, no work involving this product in ovarian follicle culture has been published so far. Thus, this study aimed to evaluate the use of PLTM as a supplement in murine pre-antral follicle culture in the context of female reproductive preservation.

## MATERIALS AND METHODS

### Ethics approval

This study was approved by the Ethics Committee in Animal Experimentation of the University of Vale Do Itajaí (UNIVALI, SC, Brazil) under No. 017/18.

### Binocular loupe

Prepubertal female mice (N=22, SWR-SWISS), aged 15-18 days, were obtained from the UNIVALI animal facility. After euthanasia of the animals in a CO_2_/O_2_ chamber, ovaries were dissected, and pre-antral ovarian follicles were mechanically isolated with two hypodermic needles (26G) in Petri dishes (Ingámed, Maringá, Brazil) and the presence of HTF-HEPES medium (Ingámed, Maringá, Brazil). This operation was performed under a binocular loupe (Olympus, Japan) and a sterile laminar flow. Follicles with intact cells, without antrum, of comparable areas were then selected for culture.

### In vitro culture of follicles

**Figure 1:**
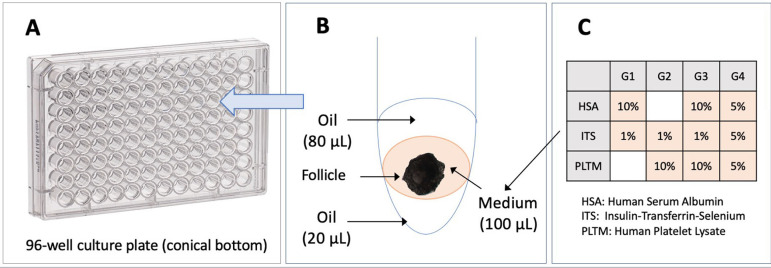
Description of the culture system. A: 96-well culture plate. B: assembly of the culture system in a well, DMEM:F12 culture medium with a follicle sandwiched between two oil layers of 20 µL (bottom layer) and 80 µL (top layer). C: protein additives to the culture medium for groups G1, G2, G3, and G4.

DMEM:F12 medium (Dulbecco’s Modified Eagle’s Medium: Nutrient Mixture Ham F-12, pH 7.32, Sigma Aldrich, São Paulo) was used for follicle culture. This essential medium was supplemented with 1% penicillin (Sigma Aldrich), 5 µg/mL streptomycin (Sigma Aldrich), and 0.5 IU/ mL equine chorionic gonadotropin (eCG, Syntex, Sigma Aldrich). Several additional supplements were tested, namely human platelet lysate (PLTMax, Sigma Aldrich), insulin, transferrin-selenium mixture (ITS, Sigma Aldrich), and human serum albumin (Ingámed). The four tested conditions (G1, G2, G3, G4) are shown in Figure 1.

The selected follicles (N=164) were cultured individually in 96-well culture plates with conical bottoms (Corning, USA). The system was based on a description made by Nikiforov *et al.* (2018) and is shown in Figure 1. Each follicle was placed in a 100 µL medium between two layers of sterilized mineral oil (Nujol, Mantecorp, Brazil) and was monitored individually from start to finish. The culture was performed in a humidified incubator at 37°C with 5% CO_2_.

After ten days of culture, the end of follicular and oocyte maturation was induced by adding 30 µL of MEM:F12 containing 1.5 IU/mL of hCG (Vetecor 5000, Hertape Callier, Brazil), previously equilibrated under 5% CO_2_. The culture was continued for another 16 hours, after which the follicles were transferred to a petri dish, photographed, and re-examined individually to determine follicular growth, vitality, and oocyte presence.

### Image acquisition and analysis

**Figure 2:**
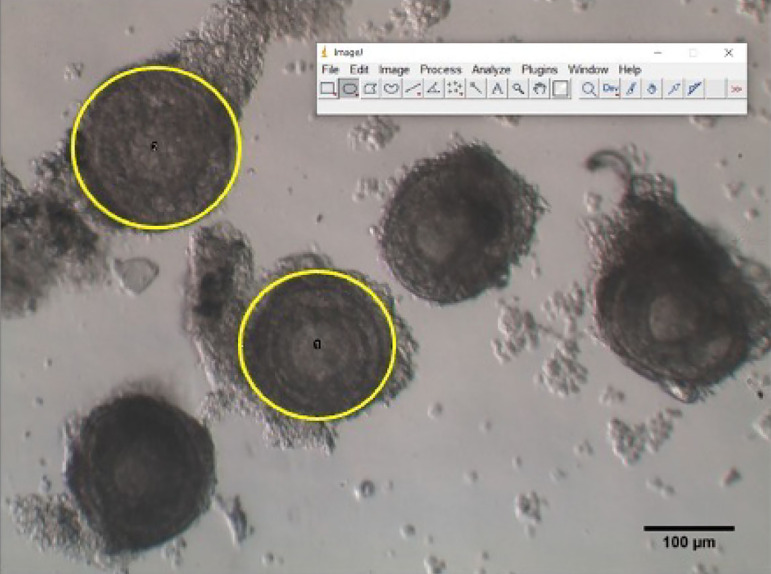
Examples of follicles recovered after dissection of prepubertal mouse ovaries. Circles in yellow correspond to the areas (expressed in µm^2^) calculated by the public domain software ImageJ.

Follicles were numbered according to their position in the culture plate and photographed using an inverted microscope (Olympus MII, Japan, 400x) equipped with an OCTAX camera and Eyeware software (Vitrolife, São Paulo). The surface of each follicle was determined with the public domain software ImageJ (NIH, Bethesda, USA). Examples of recovered follicles are shown in Figure 2.

### Assessment of follicular growth

**Figure 3:**
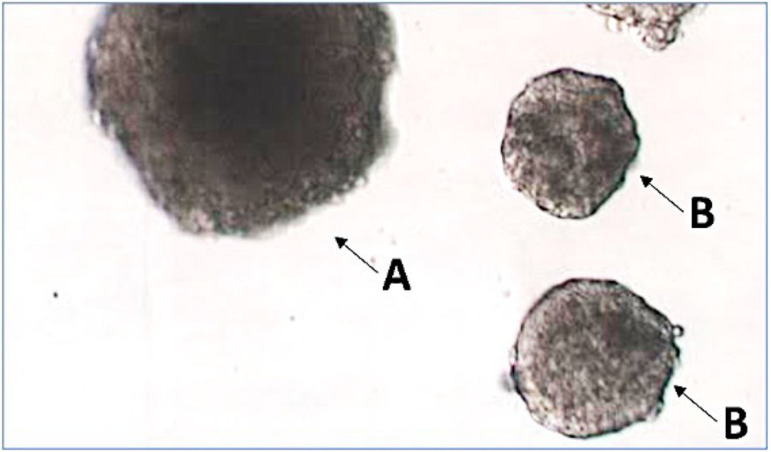
Examples of follicles after 11 days of culture and 16-18h after hCG induction of meiosis resumption. A: follicle showing mature size, B: atretic follicles that have stopped growing.

On the 11^th^ day of culture, approx. 16-18h after hCG induction, follicles were transferred one by one to a Petri dish containing HTF-HEPES medium and photographed while recording its initially established identification number. The surfaces were measured with ImageJ software (https://imagej.nih.gov/ij/download.html). Follicles with a surface area less than 19,000 µm^2^ or less than the initial surface were considered atretic (Figure 3).

### Classification of oocytes

**Figure 4:**
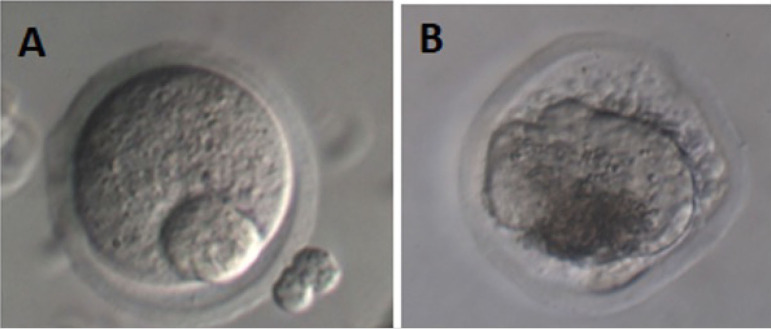
Illustration of oocytes recovered from follicles at D11. A: intact oocyte, B: atretic oocyte. A photomicrograph was obtained using an inverted microscope (Olympus MII, Japan, 400x, Hoffman Modulation Contrast). Source: Own authorship.

Approximately 16-18h after induction with hCG, the morphological quality of the obtained oocytes was evaluated, classifying them as intact or degenerated. Intact oocytes included the various stages of maturation: prophase I, metaphase I, and metaphase II. Degenerated oocytes included various forms, such as fragmented or lysed cytoplasm, meiotic cleavage, or other signs of apoptosis. See Figure 4.

### Verification of cell vitality

At the end of the cultures and analyses, the vitality of the follicular cells was assessed (Asaduzzman *et al.*, 2018). Each follicle was transferred to a microtube containing 100 µL of collagenase IV (Sigma Aldrich) and incubated for 30 min at 37°C. Then, 100 µL of hyaluronidase (80 IU/mL, Ingámed) was added for another 30 min incubation. During this procedure, the microtubes were vortexed every 15 min. Upon completion of these incubations, 50 µL of HSA (Ingámed) and 250 µL of phosphate buffer (Ingámed) were added to the microtubes, which were then centrifuged (5 min, 3500 RPM). The cell pellets were resuspended in 20 µL of phosphate buffer (Ingámed) and 20 µL of 0.4% Trypan blue (Sigma-Aldrich, USA). The cell suspensions were finally examined in a Neubauer counting chamber with a bright field microscope (400X). Unstained cells were considered alive, and stained cells were considered dead. Results were expressed as % of live forms.

### Statistical analysis

Data were analyzed with the Jamovi statistical software (version 1.6.23.0, Sydney, Australia). An ANOVA test was used to analyze continuous variables and chi-square for categorical variables. Differences were considered statistically significant for *p* values <0.05.

## RESULTS

**Figure 5:**
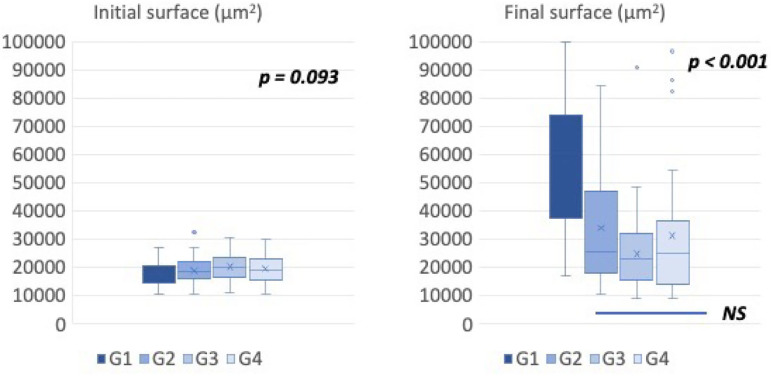
Boxplot representation of follicular surfaces in µm^2^ between the beginning (D1, left panel) and the end of the culture (D11, right panel) in the 4 conditions tested (G1: HSA 5% + ITS 1%, G2: PLTM 5% + ITS 1%, G3: HSA 10% + ITS 1% + PLTM 10%, G4: HSA 5% + ITS 5% + PLTM 5%. Statistical comparison was made using ANOVA and Post Hoc tests.

The follicular surfaces measured on the first day of culture (D1, initial surfaces) and on the 11^th^ day (D11, final surfaces) are presented in Table 1 and as box plots in Figure 5. The results are averages of three experiments in which the four conditions (G1, G2, G3, G4) were tested in parallel. At D1, follicular sizes were comparable in the four groups (ANOVA, *p*=0.09). The follicle sizes increased in all tested conditions, with the most significant increases observed in G1 (HSA+ITS) and G2 (PLTM+ITS). The mean final sizes obtained at D11 showed significant differences (ANOVA, *p*<0.001).

**Table 1:** Initial and final follicular surfaces, surface increase ratios in the four groups studied. N represents the number of follicles obtained in 3 experiments where the four conditions were tested in parallel. Statistical comparisons between initial and final follicular surfaces were made by Student’s t-test.

Groups	N	Initial surface x 10^3^ µm^2^ Mean ± SE	Final surface x 103 µm^2^ Mean ± SE	Final/initial ratio Mean ± SD	*p*
G1	33	17.94±0.69	57.27±4.04	3.4±1.9	**<.001**
G2	38	18.76±0.71	33.85±3.75	1.8±1.3	**<.001**
G3	37	20.45±0.76	24.53±2.43	1.2±0.8	0.104
G4	38	19.29±0.74	31.27±3.97	1.7±1.5	**0.006**

The initial and final sizes within each group were also compared. They were significantly different for groups G1, G2 (*p*<0.001), and G4 (*p*<0.01), while in group G3, the follicular growth was not significant (*p*=0.104) (Table 1).

**Figure 6:**
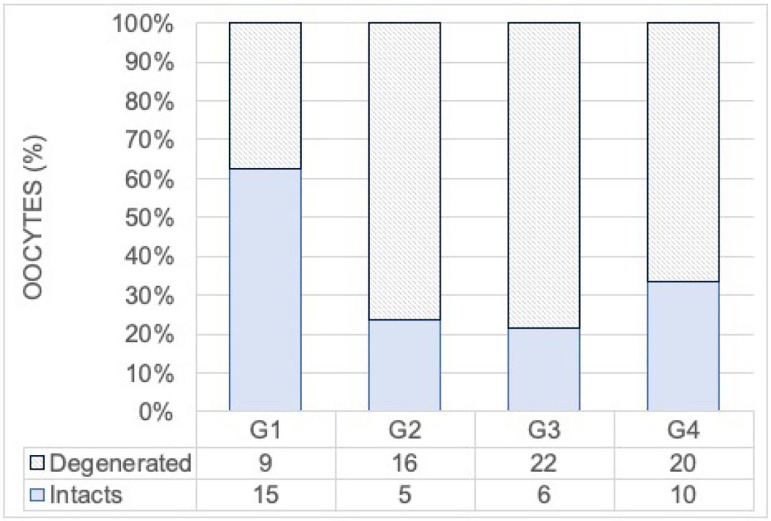
Distribution of intact and degenerated oocytes identified at the end of culture in the G1, G2, G3, and G4 groups. Comparison of groups was made by chi-square (χ^2^ (N=103,3) = 11.4, p=0.01).

In all four conditions, intact or degenerated oocytes were recovered, the total number oscillating between 21 and 30 per group (Figure 6). The G1 group stands out for a higher number of intact oocytes (>50%) compared to the others (<35%). This difference is significant (χ^2^ : (N=103,3) = 11.4, *p*=0.01)

**Figure 7:**
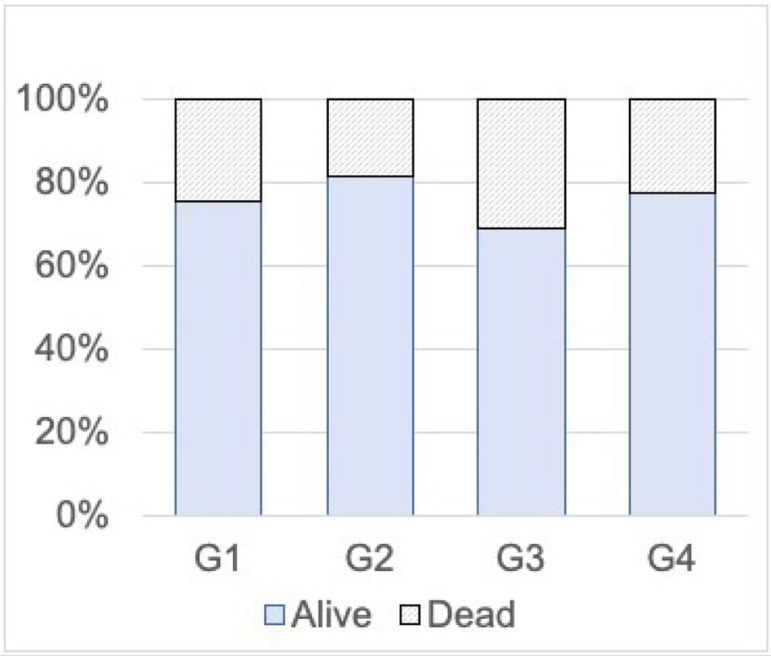
Cell vitality (%) of follicles at the end of the culture period for the four conditions tested (G1, G2, G3, G4). Statistical comparison by chi-square (χ^2^ (N=2958,3) = 19.1, p<0.01).

Finally, the cell vitality of follicles at the end of the culture and the distribution of dead/live cells are presented in Figure 7. All four groups showed vitality rates above 70%. Statistically, these rates are significantly different in a chisquare test (χ^2^: (N=2958,3) = 19.1, *p*<0.01).

## DISCUSSION

*In vitro* culture of ovarian follicles is an emerging technique in oncofertility that is becoming an alternative to the *in situ* reimplantations of ovarian fragments (Rajabzadeh *et al.*, 2020). Several approaches have been described for isolating preantral follicles from ovarian fragments and their *in vitro* culture (Pazoki *et al.*, 2015a; Figueiredo & Lima, 2017; Xu *et al.*, 2021). We chose to work with mechanically isolated follicles cultured in a medium between two layers of mineral oil. This method has an advantage over the two-dimensional culture system, as it prevents cell adhesion to the culture dish’s surface while preserving the follicle’s three-dimensional structure (Nikiforov *et al.*, 2018). This mode also ensures the homogeneous delivery of nutrients to the follicle, promotes the maintenance of intercellular junctions, and helps maintain the oocyte within the granulosa cell layers (Asaduzzman *et al.*, 2018; Heiligentag & Eichenlaub-Ritter, 2017). However, three-dimensional cultures using a gel matrix have limitations in providing nutrients to the developing tissue mass, as the substance in which the follicle is encapsulated exerts an inhibitory control on follicular growth initiation (Shea *et al.*, 2014).

Our study shows that using PLTM platelet lysate, alone or in conjunction with HSA, significantly reduces follicular growth compared to the group using HSA alone (Figure 6). The initial sizes of the collected follicles were equal in the four groups tested (*p*=0.09). The differences in size observed after 11 days of culture could not be associated with the homogeneity of the initially selected follicles. Significant follicular growth was observed in all groups except for the G3 group, which contained 10% PLTM and 10% HSA (Table 1). Follicles in this group had significantly lower cell vitality (~70%) (*p*<0.001, Figure 7) than those in other groups (~80%). The amount of protein added to this group (10% HSA, 10% PLTM) was higher than that added to the G1 (10% HSA), G2 (10% PLTM), and G4 (5% HSA and 5% PLTM) groups. This combination could have caused an inhibition of cell proliferation. Identifying factors that promote follicular development or induce atresia is one of the main objectives of research on folliculogenesis. The extrinsic and intrinsic pathways of apoptosis induction in granulosa cells are not yet clearly defined but include the absence of growth factors or cytotoxic stimuli (Demeestere *et al.*, 2005).

A study on pre-antral follicles encapsulated in fibrin gel transplanted into the subcutaneous region of rats showed after 14 days a higher survival rate in the presence of 15% platelet lysate (PL) compared to 5%, 10%, and 20% concentrations, while follicle growth and maturation did not differ from the control without PL (Rajabzadeh *et al.*, 2015). Similar results were observed in umbilical cord-derived PL, leading to lower follicle growth and estradiol secretion (Pazoki *et al.*, 2015a). However, follicles cultured in 5% PL concentrations showed the highest survival rate (93.5%), with less formation of abnormal follicles (4.1%), but without showing oocyte maturation.

In a human corneal epithelial cell proliferation study, PLTM induced more significant inhibitory effects than fetal bovine serum (FBS) supplementation (Huang *et al.*, 2017). In contrast, this effect was not observed on other cell lines, such as fibroblasts, endothelial cells, or tumor cells (Burnouf *et al.*, 2016). Thus, different cell types show distinct responses when cultured in PLTM (Kakudo *et al.*, 2019). According to our results, murine follicular cells respond negatively to PLTM, at least at the concentration of 10% used. The response might be different at other concentrations.

In our study, we observed that some immature and degenerate oocytes were released from the ovarian follicles before ending the culture period, as already described by other authors (Murase *et al.*, 2018). It was proposed that ovarian follicles cultured with PL secreted a more significant amount of progesterone than the group supplemented with SFB, which may explain the early release of immature oocytes from follicles. Since progesterone is one of the factors responsible for oocyte maturation and final ovulation, it was suggested that LP could be used *in vitro* to induce ovulation (Pazoki *et al.*, 2015a). In another study by the same group, denuded oocytes matured in a medium containing a lower concentration of hPL (5%) showed a higher maturation rate (75%) compared to the group using SFB (60%) (Pazoki *et al.*, 2015b). Our study found a higher number (*p*=0.01) of degenerated oocytes in the PLTM groups than in the group containing HSA. Although follicular cells could survive in the medium with PLTM, they may not have been able to provide the necessary elements to maintain oocyte viability. The early exit of oocytes occurred in all groups. It may thus be the result of a common cause in all four groups, such as micro-injuries caused to the follicular membrane by the mechanical isolation performed in this study. Mechanically isolated follicles show lower viability shortly after isolation. However, they provide higher growth, follicular survival, and mature oocytes after culture than enzymatic isolation (Kim *et al.*, 2018).

We observed in our study that cell proliferation and increase in the follicular area did not show homogeneity regardless of the groups tested, suggesting that many factors may mediate follicle individuality. Xu *et al.* (2010) also observed three distinct cohorts of monkey secondary follicles grown *in vitro*, namely: non-growing, slow-growing (doubling the size), and fast-growing follicles (at least 3-fold the diameter). The differences in the growth of human ovarian follicles were also demonstrated in another study by the same group (Xu *et al.*, 2009). Secondary follicles are heterogeneous in their ability to grow *in vitro*, being a possible response of the follicle to hormonal stimuli, as well as their ability to respond to and produce other growth factors (Telfer & Zelinski, 2013).

A complex interplay of extra-ovarian metabolic factors and endogenous signals regulates follicular growth. Elucidation of these control systems is one of the significant challenges to a better understanding of the processes involved in ovarian folliculogenesis and the production of viable oocytes (Martins *et al.*, 2008; Scaramuzzi *et al.*, 2011). Ovarian follicles have several paracrine factors that regulate their development; the absence of these factors can lead to the proliferation of granulosa cells without adequate nuclear and cytoplasmic maturation of the oocyte. Consequently, oocytes obtained *in vitro* may not be competent for fertilization and embryonic development (Guzel & Oktem, 2017). However, competent oocytes and births have been obtained from murine primordial follicles (O’Brien *et al.*, 2003), pre-antral vitrified murine ovarian tissue (Wang *et al.*, 2011), and in humans (Yang *et al.*, 2020; Xu *et al.*, 2021).

To our knowledge, our study has shown the use of PLTM in follicular development *in vitro* for the first time. It is an interesting supplementation candidate that deserves further exploration, especially for the concentrations used. Studies on in vitro follicular development combined with cryopreservation of pre-antral follicles are needed, as these techniques could be a real option for fertility preservation for women who must undergo immediate chemotherapy treatments and prepubertal girls.
